# Analysis of the phytochemicals of *Coriandrum sativum* and *Cichorium intybus* aqueous extracts and their biological effects on broiler chickens

**DOI:** 10.1038/s41598-022-10329-2

**Published:** 2022-04-16

**Authors:** Hanaa S. S. Gazwi, Magda E. Mahmoud, Enas M. A. Toson

**Affiliations:** 1 Department of Agricultural Chemistry, Faculty of Agriculture, Minia University, El-Minia, Egypt; 2 Department of Animal and Poultry Production, Faculty of Agriculture, Minia University, El-Minia, Egypt

**Keywords:** Nutrition, Chemical biology, Zoology

## Abstract

Spices and herbs can be used as feed additives and viable alternatives to antibiotics in chicken production. This study analyzed the phytochemicals, minerals, and antioxidant activity of aqueous extracts from *Coriandrum sativum* seeds and *Cichorium intybus* roots. The effects of different concentrations of *C. sativum* and *C. intybus* extracts on blood parameters, growth and carcass traits, biochemical parameters, and antioxidant activity of broiler chicks were also examined. The results showed that *C. sativum* aqueous extract has relatively higher contents of total flavonoids and total phenolic acids than *C. intybus* aqueous extract. Both extracts contain elevated mineral elements, especially iron, potassium, and sodium. Therefore, dietary supplementation of *C. sativum* seed and *C. intybus* root extracts could enhance broiler chicken growth performance, carcass characteristics, liver function, lipid profile, and antioxidant status. These extracts could be utilized as natural feed additives and growth promoters for broiler chickens.

## Introduction

Dietary antibiotic growth promoters significantly contributed to animal and poultry production. However, most of these antibiotics have been banned in many countries, particularly the European Union, because of public health concerns regarding their residues in animal products and the development of antibiotic resistance in bacteria^1^. Presently, Scientists are increasingly interested in discovering non-synthetic alternatives to antibiotics. Phytogenic feed additives such as herbs and spices are commonly incorporated into the diets of agricultural livestock, particularly swine and poultry, to improve flavor and palatability and thus to enhance productive performance^2^. Herbs and spices have been shown to exert potent antimicrobial properties in vitro against various pathogens and as alternative feeding strategies to replace antibiotic growth promoters^3^. Nevertheless, our knowledge regarding their modes of action and aspects of their application is still limited.

Chicory (*Cicorium intybus *L.) is regarded as a significant medicinal perennial herbaceous plant belonging to the family of Asteraceae^4^. It contains significant amounts of inulin, fructooligosaccharides, flavonoids, coumarins, as well as a wide range of vitamins^5^. Chicory has been utilized as an anti-inflammatory, antiulcerogenic, anti-hepatotoxic, depurative, digestive, alexiteric, diuretic, as well as a tonic agent^6^. Inulin, in particular, is a beneficial constituent of chicory, which has the potential to standardize appetite, as well as the metabolism of lipids to glucose^7^. Chicory has been found to enhance the development of beneficial microorganisms^8^, in addition to inhibiting the growth of pathogenic bacteria in the gut^9^. Consequently, chicory can be added to the poultry diet to control the microbiota composition of the gut and improve its integrity^9^, optimizing the performance of broiler besides health status via modifying lipid metabolism along with hypolipidemic impacts^10^.

Coriander (*Coriandrum sativum* L.) is primarily utilized due to its seeds. They are a flavoring ingredient in food industries or spice in fish, curry, bread, meat, as well as meat confections. The seeds of coriander include up to 1% essential oil. Linalool is the main component that is characterized by antioxidant^11^, antibacterial^12^, hypolipidemic^13^, and anti-diabetic properties^14^. Additionally, it is featured by appetizing as well as stimulative impacts throughout the digesting process^15^.

The aim of the current study was to evaluate the phytochemicals, in vitro antioxidant activity, and metal analysis of *C. sativum* and *C. intybus* aqueous extracts. The effects of supplementing broiler chickens with varying amounts of these extracts and their combinations on productive performance, carcass features, and physiological responses were also investigated.

## Results and discussions

### Phytochemical quantification

The chemical compounds in plants are responsible for their natural antioxidant activity, and the majority of these active compounds are natural phenols or polyphenols. Leaves, fruits, seeds, and flowers have the highest natural flavonoid and phenol contents. Increased dietary consumption of antioxidants or vegetables or fruits with antioxidant activities can improve quality of life^16^.

Table 1 demonstrates that *C. sativum* and *C. intybus* extracts have different phytochemical compounds. The aqueous extract of *C. sativum* seeds had a significantly higher content of total flavonoids (p < 0.05, Table 1) and total phenolics (36.5 ± 1.7 mg of gallic acid/g of extract) than in the aqueous extract of *C. intybus* leaves (28.16 ± 1.60 mg of gallic acid/g of extract). These results agree with Nhut et al.^17^, who reported that *C. sativum* extract has significant phenolic contents.

**Table 1:** Total phenolic and flavonoid contents of *C. sativum* and *C. intybus* aqueous extracts.

	*C. sativum*	*C. intybus*
Total phenolics (mg of gallic acid/g of extract)	36.5 ± 1.7	28.16 ± 1.60
Flavonoids (mg of quercetin/g of extract)	13.3 ± 0.8	09.01 ± 0.30

Values are means ± SEM (n = 6).

### Antioxidant activities of *C. sativum* and *C. intybus* aqueous extracts

The antioxidant activities of* C. sativum* and *C. intybus* aqueous extracts were examined using DPPH, ABTS, FRAP, metal chelation, and ORAC assays. Moreover, the DPPH test was performed to assess free radical scavenging, and the results were denoted by IC50, which is the proportion of antioxidants required to reduce the initial DPPH concentration by 50%. A low IC_50_ indicates high antioxidant efficacy, whereas trolox was used as the reference component. As depicted in Table 2, the antioxidant activity of *C. sativum* was 81.9 ± 1.02 µg mL^−1^ in aqueous extract, 105.5 ± 1.030 µg mL^−1^ in aqueous extract of *C. intybus*. Therefore, *C. sativum* has an antioxidant profile that includes chelation activity, phospholipid peroxide inhibition, radical free radical scavenging activity, hydroxyl radical, and peroxided peroxidation scavenging^18^.

**Table 2:** DPPH, ABTS^+^ scavenging, FRAP, ORAC, and metal chelation, of aqueous extracts of *C. sativum* and *C. intybus*.

	*C. sativum*	*C. intybus*
DPPH (IC50 (µg/mL)	81.9 ± 1.02	105.5 ± 1.03
ABTS (µM TE/mg of extract)	706.07 ± 16.02	636.27 ± 12.87
FRAP (µM TE/mg of extract)	102.91 ± 3.07	94.25 ± 7.46
ORAC (µM TE/mg of extract)	1398.82 ± 59.15	1067.18 ± 69.10
Metal chelating property (µMEDTA eq/mg of extract)	64.23 ± 2.1	47.15 ± 1.9

Values are means ± SD (n = 6). TE Trolox equivalent.

*C. sativum* and *C. intybus* aqueous extracts were confirmed to be rapid and efficient scavengers of ABTS radicals (Table 2). The ABTST + scavenging activity results differed substantially (P 0.05) between *C. sativum* and *C. intybus* at  706.07 ± 16.02 and 636.27 ± 12.87 µM Trolox/mg of extract, respectively. ABTS radical cation scavenging activity is a reflection of its hydrogen-donating capacity. According to Pandey et al.^18^, high molecular weight phenolics (tannins) have a high capacity to quench free radicals (ABTST+). When combined with a nutrient, these extracts can function as potential nutraceuticals.

Antioxidants act as reductants and oxidant activators. Reducing power may be a significant indicator of possible antioxidant activity. In the present study, the antioxidant capacity of *C. sativum* and *C. intybus* extracts was determined by their ability to decrease the TPTZ–Fe (III) complex to the TPTZ–Fe (II) complex (Table 2). The capacity to reduce ferric ions also indicated their significant FRAP activity. The aqueous extract of *C. sativum* had a more pronounced activity (102.91 ± 3.07 µM TE/mg of extract) than that of *C. intybus* (94.25 ± 7.46 µM TE/mg of extract).

The ORAC test is the only one among the analysis methods based on the hydrogen atom transfer mechanism, uses a biologically relevant radical^19^, and quantifies the inhibition time and degree of an antioxidant^20^. According to the ORAC values of the extracts (Table 2), *C. sativum* demonstrated the highest capacity (1398.82 ± 59.15 µM TE/mg of extract), and *C. intybus* had the lowest (1067.18 ± 69.10 µM TE/mg of extract).

Although iron is required for proper physiology, its excessive amount can induce cellular damage. If the reduced metals go through the Fenton reaction, they may generate reactive hydroxyl radicals that contribute to oxidative stress^21^. The capacity to chelate/deactivate transition metals, catalyze hydroperoxide breakdown, and facilitate Fenton-type reaction is a critical mechanism of antioxidant activity. Therefore, the extracts' iron (II) chelating activity was evaluated. Both exhibited a high capacity to chelate metal ions (Table 2). The aqueous extract of *C. sativum* (64.23 ± 2. µMEDTA eq/mg of extract) showed more high chelating ability than that of *C. intybus* (47.15 ± 1.9 µMEDTA eq/mg of extract). These findings indicate that the extracts may serve as an antioxidant by sequestering Fe (II) ions, initiating Fenton-type reactions, or engaging in metal-catalyzed hydroperoxide breakdown activities. The scavenging and chelating abilities of antioxidants are determined by the number of hydroxyl groups and their unique phenolic structure^22^.

### Elemental analysis

The presence of inorganic components is essential for the survival of bioactive chemical entities. In addition to the four-building elements, carbon, hydrogen, nitrogen, and oxygen (forming the main organic molecules), living organisms require many inorganic components to function properly. Numerous elements are fundamentally necessary for normal physiological function^23^.

Twenty-two inorganic elements with an essential role in biological activities were detected in *C. sativum* and *C. intybus* extracts (Table 3). Inductively coupled plasma–optical emission spectrometry was used to analyze these elements, which is one of the most effective techniques for performing multi-element analysis quickly and with high sensitivity.

**Table 3:** Mineral content (mg/kg) of *C. sativum* and *C. intybus* extracts.

	Calcium	Sodium	Potassium	Magnesium	Aluminum	Phosphor	Boron
*C. sativum*	68,697 ± 101.3	360.54 ± 12.7	11,200 ± 200.5	5234.11 ± 113.1	19.4 ± 1.3	702.53 ± 11.29	30.14 ± 4.1
*C. intybus*	61,634 ± 112.1	500.76 ± 13.1	12,500 ± 111.1	6211.21 ± 81.0	217.5 ± 1.7	675.32 ± 14.1	185.2 ± 2.41

	Ba	Cobalt	Cadmium	Chromium	Copper	Iron	Manganese
*C. sativum*	4.28 ± 0.65	0.20 ± 0.01	0.18 ± 0.01	12.45 ± 0.65	4.16 ± 1.2	15,630 ± 181.1	56.7 ± 7.1
*C. intybus*	11.2 ± 0.42	0.22 ± 0.02	0.21 ± 0.02	53.01 ± 0.61	6.81 ± 0.89	1317 ± 71.3	42.44 ± 3.8

	Molybdenum	Nickel	Lead	Silicium	Strontium	Vanadium	Zinc
*C. sativum*	1.699 ± 0.1	< 0.002	1.06 ± 0.1	58.88 ± 1.8	0.43 ± 0.1	0.24 ± 0.02	36.21 ± 2.1
*C. intybus*	1.093 ± 0.6	1.57 ± 0.02	2.49 ± 0.02	540.76 ± 0.11	47.91 ± 3.4	< 0.01	46.7 ± 0.89

	Selenium						
*C. sativum*	N. D						
*C. intybus*	0.32 ± 0.01						

N. D.: Not detected.

The most abundant element in the samples was calcium, followed by iron, potassium, and sodium. This element is critical for various physiological processes and, along with phosphorus, serves as a structural bone component. Calcium supplementation contributes to the prevention of bone fractures and calcium-deficient diseases^24^. Inorganic elements, calcium, was the most abundant inorganic element in *C. sativum* and *C. intybus* extracts (686,97 ± 101.3 and 616,34 ± 112.1  mg/kg, respectively).

Iron, potassium, and sodium were identified as possible nutritional components (Table 3). Iron is a critical element that contributes to hemoglobin's oxygen-carrying ability and is present in various essential enzymes, including the cytochrome p450 enzyme. The amount of iron in *C. sativum* and *C. intybus* extracts was 15,630 ± 181.1 and 1317 ± 71.3  mg/kg, respectively (Table 3).

Potassium was found in high concentrations in *C. sativum* and *C. intybus* extracts (11,200 ± 200.5  and 12,500 ± 111.1 mg/kg, respectively) and contributed to fluid balance, nerve impulses, and muscular contraction. A high-potassium diet lowers blood pressure, prevents kidney stones, water retention, osteoporosis, and protects against stroke.

Sodium is one of the beneficial elements that the body needs in trace amounts to perform regular biological functions. This substance aids in the transmission of nerve impulses, the maintenance of proper water and mineral balance, and the contraction and relaxation of muscles. The human body requires 500 mg of sodium each day. In this study, 360.54 ± 12.7  and 500.76 ± 13.1 mg/kg of sodium were detected in *C. sativum* and *C. intybus* extracts, respectively. Excess sodium intake is linked to increased risks of hypertension, stroke, and heart diseases.

Phosphorus, chromium, manganese, and strontium were among the other important trace elements detected in *C. sativum* and *C. intybus* extracts (Table 3). Phosphorus is a significant energy source for the body and is a mineral that is necessary for the formation of bones, teeth, DNA, RNA, and cell membrane. Phosphorylation is included in various carbohydrates and proteins found in the body. In this study, phosphorus was detected in significant amounts in *C. sativum* and *C. intybus* extracts (702.53 ± 11.29 and 675.32 ± 14.1 mg/kg, respectively). Se, known for its ability to protect against oxidative damage^25^, was found in high concentrations (0.32 ± 0.01 mg/kg) in *C. intybus* but was not detected in *C. sativum*.

Copper is the third most prevalent trace element (after zinc and iron) in the human body and is a critical component of oxygen-transporting blood cells^26^, bones, the heart, the brain, connective tissues, and other body organs^27^. Copper concentrations were high in all tested plant materials, particularly in *C. intybus* plant sample (6.81 ± 0.89 mg/kg) (Table 3).

Zinc concentration was high in *C. intybus* (46.7 ± 0.89 mg/kg) but low in *C. sativum (*36.21 ± 2.1 mg/kg). Zinc is a trace element or micronutrient required to foe the growth and development of microbes, animals, and plants. This element is essential for protein synthesis and DNA and collaborates with copper as a cofactor in several crucial enzyme systems^28^.

In addition to these basic elements, bromine, lead, cadmium, and barium were found in the extracts of *C. sativum* and *C. intybus*. These inorganic elements are hazardous, though their concentrations were within the acceptable range.

Cadmium and lead have permissible limits of 0.3 and 10 mg/kg, respectively^29^. Table 3 lists the additional elements detected in trace amounts.

Based on these results, *C. sativum* and *C. intybus* can be recommended as raw or processed supplements for food products.

### Growth performance

Table 4 lists the average body weight gain, feed consumption, feed conversion ratio, and mortality rate of broiler chicks fed with diets supplemented with 1000 mg of *C. sativum*, 500 mg of *C. intybus*, or a mixture of  500 mg of *C. sativum* plus 250 mg of *C. intybus* extracts/kg of diet during starter, finisher, and entire experimental periods. The inclusion of *C. sativum* and *C. intybu*s extracts in broiler chick diets significantly improved body increase weight during starter, finisher, and entire periods. This finding could be attributed to the presence of antioxidants and phenolic substances in *C. sativum* and *C*. *intybus* extracts that enhanced growth performance. Jang^30^ adding coriander oil to broiler diets contributed significantly to increasing weight. Comparable findings were revealed by Faramarzzadeh et al.^31^, who added 4.5% of chicory powder to the broiler diet.

**Table 4:** Effect of the addition of *C. sativum* and *C. intybus* extracts on the growth performance of broiler chicks.

Items	Treatments				SEM	P values
	Control	1000 mg of *C. sativum*	500 mg of *C. intybus*	500 mg of *C.* *sativum* + 250 mg of *C. intybus*		
**Body weight gain**						
0–3 weeks	737.0^b^	824.0^a^	822.0^a^	779.0^b^	8.97	0.002
4–6 weeks	1398.1^c^	1633.4^a^	1626.7^a^	1526.6^b^	14.56	0.001
0–6 weeks	2121.2^c^	2442.3^a^	2433.4^a^	2331.0^b^	18.54	0.001
**Feed consumption**						
0–3 weeks	1090.9	1097.5	1108.8	1113.9	10.48	0.290
4–6 weeks	3435.8	3389.8	3443.3	3520.0	24.60	0.336
0–6 weeks	4551.3^b^	4509.3^b^	4655.6^a^	4590.2^ab^	24.76	0.009
**Feed conversion**						
0–3 weeks	1.48^a^	1.33^c^	1.33^c^	1.43^a^	0.022	0.005
4–6 weeks	2.46^a^	2.07^c^	2.16^b^	2.20^b^	0.025	0.001
0–6 weeks	2.12^a^	1.82^c^	1.89^c^	1.95^b^	0.018	0.001
Mortality rate %	6.67^a^	3.34^b^	3.34^b^	3.34^b^	0.288	0.050

^a,b^Within the same rows, means have similar letter (s) are not significant different at ≤ 0.05.

Consumption of feed during starter and finisher periods was not significantly affected by the supplementation of *C. sativum* and *C. intybus* extracts in the broiler chick diet. Meanwhile, the addition of 500 mg of *C. intybus* extract/kg of diet significantly increased feed consumption during the entire experimental period (0–6 weeks). This increase could be attributed to the phytogenic feed additives that improved the flavor and palatability of feeds^32,33^. The improvement in feed consumption induced by the powder of coriander seeds can be due to essential oils as well as their primary ingredient, which is linalool, in coriander seeds. Çabuk et al.^34^ illustrated that linalool has an appetizing impact on diets and promotes the digestion process in animals. In addition, previous studies have reported the positive impacts of essential oils on feed intake^35^.

Dietary supplementation of *C. sativum* and *C. intybus* extract significantly improved the ratio of feed conversion during all three periods. This improvement could be attributed to the appetite-enhancing, digestive-stimulating, and antimicrobial effects of the two extracts^36^. According to the present findings, the fed diet of hens supplied with chicory powder is characterized by a substantially diminished feed conversion ratio than the unsupplemented group^34^. Similarly, Cabuk et al.^34^ noted a considerable increase in the conversion ratio of feed in the diet of broilers supplied with a blend of herbal plants. Additionally, Liu et al.^37^ demonstrated that supplementing a baseline diet with chicory powder induced a considerable improvement in the consumption of feed during the first 13 days of the feeding interval, which is consistent with the current study results. Improved broiler development performance on a diet supplied with the chicory powder might be attributable to the increase in number, length, as well as the surface area of intestinal villi that are paralleled with an enhanced absorptive and digestive ability of jejunum^38^.

Furthermore, adding chicory powder to diets will benefit younger broiler chicks more than older ones owing to the stimulation of villi development^37^. The findings of Al-Jaff^39^ are consistent with the present study results. The addition of coriander seed to broiler chickens' diet induced an improvement in FCRs, due to the active component (linalool) in coriander, causing greater efficiency in feed utilization, resulting in enhanced growth^40^. Morover, Przybilla and Weiss^41^ illustrated that the herbal blend's route of action feed conversion occurs via enhancing the digestive processes. According to Rajeshwari and Andallu^42^, coriander is an efficient appetizer and contributes to the production of enzymes as well as digestive juices in the stomach, stimulating peristaltic motion and digestion, and consequently enhancing FCR.

Dietary supplementation of these extracts significantly decreased the mortality rate of broiler chicks during the experimental period. This finding could be attributed to the positive results of the two extracts for tannins, alkaloids, terpenoids, flavonoids, and saponins and the negative for glycosides. Adisa et al.^36^ indicated that tannins possess antiviral and antibacterial activities. Taraz et al.^43^ concluded that coriander seeds inhibit pathogenic microorganisms in the digestive system, decreasing birds' mortality or disease infection.

The improved growth performance of broilers fed with both extracts could be attributed to coriander. Chicory extracts have some components with antimicrobial properties, such as flavonoids and phenolics (Table 1). Deans and Waterman^44^ concluded that the addition of plant extracts to poultry diets could enhance the growth performance by stimulating endogenous enzymes and regulating intestinal microflora balance.

### Carcass characteristics

Table 5 depicts the carcass traits of broiler chicks fed with diets supplemented with 1000 mg of *C. sativum*, 500 mg of *C. intybus*, or 500 mg of *C. sativum*  + 250 mg of *C. intybus* extracts/kg of diet. These results indicated that dietary supplementation with *C. sativum* and *C. intybus* extracts significantly increased liver and carcass weight and decreased abdominal fat percentage. In contrast, the other carcass organs were not significantly affected. These findings are in agreement with prior studies^45^. The increased dressing percentage may be linked to the stimulatory effects of coriander on pancreatic secretions, which in turn boost digestive enzyme secretion and produce additional nutrients, such as amino acids that are digested and absorbed from the digestive system^46^. Generally, the accumulation of body fats can be the net result of the balance between fat catabolism, fat synthesis (lipogenesis), as well as dietary absorbed fat via β-oxidation (lipolysis).

**Table 5:** Effect of *C. sativum* and *C. intybus* extracts supplementation to diet on carcass characteristics of broiler chicks.

Items	Extracts (mg/Kg diet)				SEM	P-values
	Control	1000 mg of *C. sativum*	500 mg of *C. intybus*	500 mg of *C. sativum* + 250 mg of *C. intybus*		
Live body weight	2190.2^c^	2375.4^a^	2400.0^a^	2300.5^b^	16.24	0.001
Carcass weight	1609.0^c^	1749.7^a^	1768.6^a^	1688.8^b^	12.89	0.001
Dressing %	73.47	73.66	73.69	73.41	0.14	0.492
Liver %	2.97	2.65	2.52	2.64	0.09	0.434
Gizzard %	1.61	1.68	1.66	1.66	0.07	0.890
Heart %	0.51	0.56	0.60	0.58	0.001	0.253
Abdominal fat %	1.00^a^	0.87^a^	0.77^b^	0.66^b^	0.01	0.011

^a,b^Within the same rows, means have similar letter (s) are not significant different at ≤ 0.05.

Consequently, when the quantity of ingested fat remains constant, reduced deposition of body fats can be due to diminished synthesis of fatty acids, elevated fat catabolism, or both mechanisms. Hence, carcass characteristics are enhanced. Antioxidants and phenolic substances in herbous extracts enhanced the broiler chicken's carcass reaching 1.2%^47^.

Panda et al.^48^ and Faramarzzadeh et al.^31^ demonstrated marked improvement in the proportion of dressing by supplementation of chicory powder (3.0%) to broiler diet. The current findings also agree with Yusrizal and Chen^49^, who illustrated that the fed diets of broiler chickens supplied with chicory fructans (1%) were heavier than controls. In addition, they detected a positive correlation between the addition of chicory supplementation as well as carcass characteristics in poultry species^50^.

### Hematological parameters

Table 6 presents the effects of supplementing the broiler chick diet with 1000 mg of *C. sativum*, 500 mg of *C. intybus*, or 500 mg of *C. sativum*  + 250 mg of *C. intybus* extracts/kg of diet on hematological plasma parameters. These results revealed that this supplementation significantly increased RBCs, hemoglobin concentration, and hematocrit percentage. In contrast, WBC: heterophil and heterophil: LYM ratios were significantly reduced at six weeks of age, while LYM and MON percentages were not significantly affected. Khubeiz and Shirif^51^ reported that adding coriander seed powder to broiler chick diets significantly reduced heterophil percentage and heterophil, whereas the LYM ratio significantly increased the LYM percentage. The increase in the H/L ratio indicated that the birds were under acute stress. In the present work, the addition of *C. sativum* and *C. intybus* extracts to the broiler chick diet improved the hematological parameters at 6 weeks of age.

**Table 6:** Effect of the dietary supplementation of *C. sativum* and *C. intybus* extracts on the plasma hematological parameters of broiler chicks.

Items	Extracts (mg/kg of diet)				SEM	P-values
	Control	1000 mg of *C. sativum*	500 mg of *C. intybus *	500 mg of *C. sativum* + 250 mg of *C. intybus*		
WBCs (10^3^/µL)	54.79^a^	52.35^a^	50.66^b^	49.68^b^	2.48	0.042
LYM (L)%	72.16	72.88	74.96	75.37	5.88	0.275
Heterophil (H)%	18.98^a^	16.65^b^	16.68^b^	16.31^b^	0.97	0.045
Monocyte %	8.86	9.47	8.36	8.32	0.09	0.638
H/L ratio	0.26^a^	0.23^b^	0.22^b^	0.22^b^	0.03	0.051
RBCs (10^6^/µL)	2.98^ab^	3.00^ab^	2.93^b^	3,08^a^	0.08	0.018
Hb (g/dL)	12.11^d^	16.11^c^	17.61^b^	19.81^a^	0.71	0.001
HCT%	23.46^b^	27.54^a^	25.07^ab^	26.33^a^	0.29	0.032

^a,b^Within the same rows, means have similar letter (s) are not significant different at ≤ 0.05.

### Liver and kidney function

The effects of supplementing the broiler chick diet with 1000 mg of *C. sativum*, 500 mg of *C. intybus*, or 500 mg of *C. sativum*  + 250 mg of *C. intybu*s extract/kg of diet on **t**otal protein, albumin, globulin, A/G ratio, urea uric acid, AST, and ALT are depicted in Table 7. These results indicate that this supplementation did not significantly affect total plasma protein, albumin globulin, and A/G ratio.

**Table 7:** Effect of the dietary supplementation of *C. sativum* and *C. intybus* extracts on the plasma liver and kidney function of broiler chicks.

Items	Extracts (mg/ kg of diet)				SEM	P-values
	Control	1000 mg of *C. sativum*	500 mg of *C. intybus*	500 mg of *C. sativum* + 250 mg of *C. intybus*		
Total protein (mg/dl)	4.53	4.60	4.42	4.31	0.10	0.381
Albumin (mg/dl)	2.40	2.53	2.29	2.31	0.08	0.504
Globulin (mg/dl)	2.13	2.07	2.13	2.00	0.07	0.412
A/G	1.13	1.22	1.08	1.16	0.05	0.325
AST (U/ml)	41.48^b^	43.39^ab^	45.35^a^	41.39^b^	4.12	0.031
ALT (U/ml)	24.40^b^	27.53^a^	29.17^a^	24.30^b^	2.98	0.005
Uric acid (mg/dl)	0.46^a^	0.26^b^	0.19^b^	0.14^b^	0.01	0.048
Urea (mg/dl)	4.42^a^	3.94^b^	4.15^b^	4.10^b^	0.24	0.051

^a,b^Within the same rows, means have similar letter(s) are not significant different at ≤ 0.05.

Plasma AST and ALT were significantly affected by the dietary addition of the two extracts (Table 7)**.** Similar results were obtained by Al-Jaff^39^, who reported that supplementing broiler diets with coriander seeds significantly decreased AST and ALT. This reduction in plasma AST and ALT indicated that plant extracts do not negatively affect liver function and have no adverse influence on the growth and health of broiler chicks.

Table 7 shows that plasma urea and uric acid concentrations in broiler chicks were significantly decreased when fed with a diet supplemented with *C. sativum* and *C. intybus* extracts. Therefore, adding *C. sativum* and *C. intybus* extracts to the broiler chick diet did not adversely affect kidney function, and the extracts do not have any toxic substances.

These findings support the hepatoprotective impact of *C. intybus* and *C. sativum* extracts, suggesting enhanced kidney and liver functions, which is consistent with Khubeiz and Shirif et al.^51^ and Hosseinzadeh et al.^52^. Aromatic plant supplements in the chicken are characterized by stimulatory impacts on the digesting process by increasing digestive enzymes production and enhancing the use of digestive products through an improved liver function^52^.

### Lipid profile and glucose

Table 8 shows the average levels of plasma TC, TG, LDL, HDL, and Glucose of the broiler chicks supplemented with 1000 mg of *C. sativum*, 500 mg of *C. intybus*, or 500 mg of *C. sativum*  + 250 mg of *C. intybus* extracts/kg of diet. The results showed that this supplementation significantly decreased plasma TC, TG, and LDL. *C. sativum* and *C. intybus* extracts contain flavonoids that improve lipid metabolism in broiler chicks^53^. Coriander oil acts as a reversible competitive inhibitor of the HMG-CoA reductase enzyme, thereby inhibiting mevalonate synthesis and causing hypocholesterolemia^54^. The results obtained are compatible with those of Faramarzzadeh et al.^31^, Yusrizal and Chen^49^, Agazadehh et al.^55^, and Khoobani et al.^56^, who demonstrated that chicory-based fructan addition to the broiler diet induced a decline in serum total cholesterol, as well as LDL and triglycerides in broilers. This finding is consistent with Elson^57^, who illustrated that these isoprenoids inhibit the synthesis of cholesterol by suppressing 3-hydroxy-3-methylglutaryl coenzyme A (HMG-CoA) reductase production, the rate-controlling enzyme of the synthetic cholesterol pathway. At the same time, adding chicory extract resulted in alleviated cholesterol absorption reaching 41% (P < 0.05) in the ileum as well as 30% (P < 0.05) in the jejunum compared to controls^58^.

**Table 8:** Effect of *C. sativum* and *C. intybus* extracts supplementation to diet on plasma lipid profile and glucose of broiler chicks.

Items	Extracts (mg/ kg of diet)				SE	P value
	Control	1000 mg of *C*. *sativum*	500 mg of *C. intybus*	500 mg of *C. sativum* + 250 mg of *C. intybu*s		
Cholesterol (mg/dl)	151.4^a^	143.1^b^	140.8^b^	138.2^b^	9.12	0.041
Triglycerides (mg/dl)	82.7^a^	64.11^b^	52.4^b^	57.1^b^	8.27	0.021
HDL (mg/dl)	75.4^a^	79.8^ab^	87.1^b^	88.4^b^	5.33	0.032
LDL (mg/dl)	64.8^a^	60.4^a^	30.6^b^	43.5^b^	9.17	0.025
Glucose (mg/dl)	199.4^a^	179.2^b^	162.8^b^	149.9^c^	6.20	0.020

^a,b^Within the same rows, means have similar letter(s) are not significant different at ≤ 0.05.

Glucose concentration was significantly decreased upon the supplementation of *C. sativum* and *C. intybus* extracts in the diet of broiler chicks at six weeks of age (Table 8). The present paper's results are consistent with those of Khoobani et al.^56^, who revealed that the addition of chicory to the broiler diet resulted in diminished serum glucose. Hosseinzadeh et al.^52^ reported that coriander extracts a significant decrease in serum glucose in broilers in the control group. The decrease in glucose concentration might be attributed to the action of coriander in stimulating insulin secretion and enhancing glucose uptake and metabolism by the muscle^42^.

### Antioxidant activity

Table 9 depicts the effects of dietary supplementation with different levels of extracts (1000 mg of *C. sativum*, 500 mg of *C. intybus*, or 500 mg of C. *sativum*  + 250 mg of *C. intybus* ) on SOD, MDA, TAC, plasma GSH, and CAT levels in broiler chicks. SOD was significantly decreased, and TAC was significantly increased. MDA, plasma GSH, and CAT were not significantly influenced by extract supplementation. A high plasma antioxidant capacity was obtained upon supplementation of 500 mg of *C. sativum*  + 250 mg of *C. intybus* extracts/ kg of diet.

**Table 9:** Effect of *C. sativum* and *C. intybus * extracts supplementation to diet on antioxidant status of broiler chicks.

Items	Extracts (mg/ kg of diet)				SE	P value
	Control	1000 mg of *C. sativum*	500 mg of *C. intybus*	500 mg of *C*. *sativum* + 250 mg of C. intybus		
SOD (U/ml)	41.81^c^	48.4^b^	53.54^ab^	58.08^a^	1.45	0.052
MAD (mmo/ml)	5100	52.38	54.58	53.65	2.08	0.568
TAC (U/ml)	0.75^b^	0.80^b^	0.77^b^	1.21^a^	0.05	0.051
GSH (µg/ml)	72.42	77.65	74.92	75.66	2.18	0.468
CAT (µg/ml)	314.2	309.6	2.89.9	2.84.7	21.8	0.652

^a,b^Within the same rows, means have similar letter (s) are not significant different at ≤ 0.05.

Modifications in antioxidant enzyme parameters, as well as a decline in some nonenzymatic antioxidants, may induce oxidative stress. In addition, Natural biological processes constantly generate reactive oxygen species, neutralized by the antioxidant enzymes in the human body, such as SOD and GSH-PX, besides certain protective substances^59^ The antioxidant state of poultry may be determined by testing four main antioxidant enzymes: GSH-PX, T-AOC, MDA, and T-SOD. Total superoxide dismutase and GSH-PX are natural scavengers to free radicals by the conversion of oxygen radicals to hydrogen peroxide in order to alleviate the damaging reactive oxygen species from the cellular environment^60^. Malondialdehyde has been frequently used as an indicator to assess lipid peroxidation in biological processes; nevertheless, it is observed that it is correlated with free radical damage along with increased varying diseases^61^. In general, antioxidant defense systems, like the nonenzymatic and enzymatic systems, can be indicated by T-AOC, with elevated T-AOC in broiler chicks supplied with *C. intybus* and *C. sativum* extracts, reflecting enhanced antioxidant defense. Ahmed^62^ reported that supplementing the broiler chick diet with plant products with high antioxidant compounds contents could improve the reactive oxygen scavenging activity and conserve fat sources.

## Conclusion

This study demonstrated that *C. intybus* and *C. sativum* extract enhanced broiler chicks' performance indices, hematobiochemical profiles, as well as carcass characteristics. In addition, they improve animal health and well-being through their contribution to the antioxidant defense mechanism against free radical generation. Consequently, it is evident that these two herbs (*Cichorium intybus* and *Coriandrum sativum*) can improve performance without inducing any adverse effects on studied features.

## Materials and methods

### Extracts preparation

*C. sativum* seeds and *C. intybus* roots were purchased from a local market, dried, separately ground to a fine powder, and added 500 mL of distilled water to prepare *C. sativum* and *C. intybus* extracts. The mixture was heated for 30 min in a water bath at 65 °C after 24 h of maceration at room temperature. Following filtration, the extracts were concentrated using a rotary evaporator and stored at 4 °C for later use. The collection of herbs complies with relevant institutional, national, and international guidelines.

### Estimation of total flavonoid content and total phenolic content

Total phenolic content was measured using Folin–Ciocalteu colorimetric technique^63^ and expressed using gallic acid equivalents (mg gallic acid/g of extract). Colorimetry with aluminum chloride determined total flavonoid content^64^ and expressed using quercetin equivalents (mg of quercetin/g of extract).

### In vitro antioxidant activity of extracts

Antioxidant capacity was evaluated using the assays of scavenging free radicals (ABTS and DPPH), ferric-reducing antioxidant power (FRAP), oxygen radical antioxidant capacity (ORAC), and metal chelation, which was performed following the methods of Brand-Williams et al.^65^ for DPPH, Benzie et al.^66^ for FRAP, Arnao et al.^67^ for ABTS, Chew et al.^68^ for metal chelation, and Liang et al.^69^ for ORAC.

### Elemental analysis by ICP-MS

Inductively coupled argon plasma (ICAP 6500 Duo, Thermo Scientific, England) was used to identify the components in the extracts and digests. The stock solution for instrument standardization was a 1000 mg/L multi-element certified standard solution from Merck, Germany. Leggett and Westermann's technique^70^ was followed for sample digestion.

### Animal welfare

The committee of the Poultry Production Department at Faculty of Agriculture, Minia University, approved the current research procedures. These procedures recommend animal rights and welfare by assuring minimal stress to animals. All methods were carried out in accordance with relevant guidelines and regulations. The study was carried out in compliance with the ARRIVE guidelines.

### Growth performance trial

A total of 240-day-old unsexed broiler chicks (Ross 308) were randomly divided into 60 birds per treatment group. Each group was randomly allocated into five replicates (12 chicks each), kept in a wire cage (100 × 50 × 50 cm), and provided with a feeder and drinker. For the first 3 days, the brooding temperature was set at 34 °C and then decreased gradually to 24 °C at the end of the experimental period. The chicks were reared under a continuous program with 24 h of light during the 1st week and 23 h of light and 1 h of darkness for the remaining experimental period. The first group was fed on a basal diet (control), and the remaining groups were fed on a basal diet supplemented with 1000 mg of *C. sativum* (2nd group), 500 mg of *C.* *intybus* (3rd group), and 500 mg of *C. sativum*  + 250 mg *C. intybus* extracts/kg of diet (4th group). All broiler chicks were fed with starter (0–3 weeks) and finisher (4–6 weeks) diets formulated according to the National Research Council (NRC, 1994). Table 10 presents starter and finisher diets' ingredients and chemical composition. Feed and water were supplied ad libitum during the experimental period. The birds were vaccinated against Newcastle disease at 1 and 18 days of age and Gumboro disease at 14 and 24 days of age. Bodyweight gain feed consumption and feed conversion ratio were measured during starter (0–3 weeks), finisher (4–6 weeks), and whole experimental periods (0–6 weeks).

**Table 10:** Ingredients and chemical composition of the starter and finisher diets of broiler chicks.

Ingredients	Starter diet	Finisher diet
Yellow corn	54.35	61.85
Soy bean meal (44% CP%)	30.00	22.70
Concentrate mixture	10.00	10.00
Vegetable oil	2.20	2.20
Dicalcium phosphate	2.00	1.80
Calcium carbonate	0.80	0.80
Premix	0.25	0.25
Methionine	0.25	0.25
Lysine	0.10	0.10
Total	100	100
**Calculated chemical analysis**		
Digestible protein (%)	22.69	20.13
Metabolizable energy (Kcal/kg)	3030	3150
Calcium (%)	1.00	0.98
Available phosphorus (%)	0.54	0.49
Lysine (%)	1.24	1.18
Methionine (%)	0.60	0.58

### Carcass traits and blood samplings

Ten birds were selected from each experimental group at six weeks of age and hand slaughtered. Carcass, liver, gizzard heart, and abdominal fat were removed and individually weighed, and values were expressed as percentages of preslaughter live weight. Blood was collected at slaughter time in heparinized tubes to determine the following hematological parameters: red blood cells (RBCs), hemoglobin concentration (Hb), hematocrit (HC), white blood cells (WBCs), lymphocytes (LYMs), monocytes (MONs), and heterophil. A total of 100 leukocytes for each bird were counted to calculate heterophil: MONand heterophil: LYM ratios. Blood samples were centrifuged at 3000 rpm for 15 min. The plasma was maintained at − 20 °C until the estimation of the following biochemical parameters using commercial colorimetric kits (Biodiagnostic Company, Giza, Egypt): total protein, albumin, globulin, alanine transaminase (ALT), aspartate transaminase (AST), urea, uric acid, total cholesterol (TC), and triglyceride (TG). Plasma total antioxidant capacity (TAC), malondialdehyde (MDA), superoxide dismutase (SOD), catalase (CAT), and glutathione (GSH) were determined using commercial kits and a spectrophotometer (Shimadzu, Japan).

### Statistical analysis

Data were assessed by one-way ANOVA in the SAS Institute. Differences between means at p < 0.05 were compared using Duncan's multiple range test.

## Data Availability

The datasets utilized and analyzed during this investigation are available upon reasonable request from the corresponding author.

## References

[1] Lee KW, Everts H, Beynen AC (2004). Essential oils in broiler nutrition. Int. J. Poult. Sci..

[2] Windisch W, Schedle K, Plitzner C, Kroismayr A (2008). Use of phytogenic products as feed additives for swine and poultry. J. Anim. Sci..

[3] Lee SH, Lillehoj HS, Jang SI, Lillehoj EP, Min W, Bravo D (2013). Dietary supplementation of young broiler chickens with capsicum and turmeric oleoresins increases resistance to necrotic enteritis. Br. J. Nutr..

[4] Ivarsson E, Frankow-Lindberg BE, Andersson HK, Lindberg JE (2011). Growth performance, digestibility and faecal coliform bacteria in weaned piglets fed a cereal-based diet including either chicory (*Cichorium intybus*) or ribwort (*Plantago lanceolata*) forage. Animal.

[5] Bischoff TA, Nguyen-Dinh P, Arefiu AG, Laurantos M, Kelley CJ, Karchesy Y (2004). Antimalarial activity of Lactucin and Lactucopicrin: Sesquiterpene lactones isolated from *Cichorium intybus* L. J. Ethnopharmacol..

[6] Nandagopal S, Kumari BDR (2007). Phytochemical and antibacterial studies of chicory (*Cichorium intybus* L.)—A multipurpose, & medicinal plant. Adv. Biol. Res..

[7] Lunn J, Buttriss JL (2007). Carbohydrates and dietary fiber. Nutr. Bull..

[8] Xu ZR, Hu CH, Xia MS, Zhan XA, Wang MQ (2003). Effects of dietary fructooligosaccharide on digestive enzyme activities, intestinal microflora and morphology of male broilers. Poult. Sci..

[9] Flickinger EA, Van Loo J, Fahey GC (2003). Nutritional responses to the presence of inulin and oligofructose in the diets of domesticated animals: A review. Crit. Rev. Food Sci. Nutr..

[10] Azorin-Ortuno M, Urban C, Ceron JJ, Tecles F, Allende A, Barberan FA (2009). Effect of low inulin doses with different polymerization degree on lipid metabolism, mineral absorption, and intestinal microbiota in rats with fat-supplemented diet. Food Chem..

[11] Kubo I, Fujita KI, Kubo A, Nihei KI, Ogura T (2004). Antibacterial activity of coriander volatile compounds against *Salmonella choleraesuis*. J. Agric. Food Chem..

[12] Wangensteen H, Samuelsen AB, Malterud KE (2004). Antioxidant activity in extracts from coriander. Food Chem..

[13] Gallagher AM, Flatt PR, Duffy G, Abdel-Wahab YH (2003). The effects of traditional antidiabetic plants on in vitro glucose diffusion. Nutr. Res..

[14] Chithra V, Leelamma S (2000). *Coriandrum sativum*—effect on lipid metabolism in 1,2-dimethyl hydrazine induced colon cancer. J. Ethnopharmacol..

[15] Çabuk, M., Alçiçek, A., Bozkurt, M. & Imre, N. Antimicrobial properties of the essential oils isolated from aromatic plants and using possibility as alternative feed additives. II. In *National Animal Nutrition Congress; Konya, Turkey* Vol. 18, 184–187 (2003).

[16] Siriwardhana SSKW, Shahidi F (2002). Antiradical activity of extracts of almond and its by-products. J. Am. Oil Chem. Soc..

[17] Nhut, P. T., Quyen, N. T. N., Truc, T. T., Minh, L. V., An, T. N. T. & Anh, N. H. T. Preliminary study on phytochemical, phenolic content, flavonoids and antioxidant activity of *Coriandrum sativum *L. originating in Vietnam. In *IOP Conf. Series: Materials Science and Engineering* Vol. 991, 012022, 10.1088/1757-899X/991/1/012022 (IOP Publishing, 2020).

[18] Pandey A, Bigoniya P, Raj V, Patel KK (2011). Pharmacological screening of *Coriandrum sativum* Linn. for hepatoprotective activity. J. Pharm. Bioallied Sci..

[19] Dudonne S, Vitrac X, Coutiere P, Woillez M, Merillon JM (2009). Comparative study of antioxidant properties and total phenolic content of 30 plant extracts of industrial interest using DPPH, ABTS, FRAP, SOD, And ORAC assays. J. Agric. Food Chem..

[20] Ou B, Hampsch-Woodill M, Prior RL (2001). Development and validation of an improved oxygen radical absorbance capacity assay using fluorescein as the fluorescent probe. J. Agric. Food Chem..

[21] Hippeli S, Elstner EF (1999). Transition metal ion-catalyzed oxygen activation during pathogenic processes. FEBS Lett..

[22] Pazos M, Gallardo JM, Torres JL, Medina I (2005). Activity of grape polyphenols as inhibitors of the oxidation of fish lipids and fish muscle. Food Chem.

[23] Colotti G, Ilari A, Boffi A, Morea V (2013). Metals and metal derivatives in medicine”. Mini-Rev. Med. Chem..

[24] Bauer DC (2013). Calcium supplements and fracture prevention. N. Engl. J. Med..

[25] Patching SG, Gardiner PH (1999). Recent developments in selenium metabolism and chemical speciation: a review. J. Trace Elem. Med. Biol..

[26] Kaur R (2012). Investigation of major and trace elements in some medicinal plants using PIXE. Int. JPIXE..

[27] Morabad RB, Patil SJ, Tapash RR (2013). First series transition elemental analysis in some therapeutically important medicinal plants by AAS method. J. Mater. Environ. Sci..

[28] Gaëlle P, Amélie G, Julie P, Mourad S, Charles MD (2013). Iron, copper, zinc, and manganese transport and regulation in pathogenic Enterobacteria: Correlations between strains, site of infection and the relative importance of the different metal transport systems for virulence. Front. Cell. Infect. Microbiol..

[29] John S, Qin Q, Yukio K, David Y, Yueming J (2005). Stability and synergistic effect of antioxidative properties of lycopene and other active components. Crit. Rev. Food Sci. Nutr..

[30] Jang JP (2011). Effect of different levels of coriander oil on performance and blood parameters of broiler chickens. Ann. Biol. Res..

[31] Faramarzzadeh M, Behroozlak M, Samadian F, Vahedi V (2017). Effects of chicory powder and butyric acid combination on performance, carcass traits and some blood parameters in broiler chickens. Iran. J. Appl. Anim. Sci..

[32] Windisch W, Schedle K, Plitzner C, Kroismayr A (2008). Use of phytogenic products as feed additives for swine and poultry. J. Anim. Sci..

[33] Jemroz, D., Werlecki, T.I., Wiltckidewiez, A. & Skorapinska, J. Influence of phytogenic extracts on gut microbial status in chickens. In *Proceeding of the 14th European Symposium on Poultry Nutrition Lillehammer Norway* Vol. 176. 10.22358/jafs/67752/2003 (2002).

[34] Çabuk, M., Alçiçek, A., Bozkurt, M. & İmre, N. Antimicrobial properties of the essential oils isolated from aromatic plants and using possibility as alternative feed additives. II. National Animal Nutrition Congress. 18–20 September, Konya, Turkey, 184–187 (2003).

[35] Williams P, Losa R (2001). The use of essential oils and their compounds in poultry nutrition. World Poult..

[36] Adisa RM, Choudhary EA, Adenoye GA, Olorunsogo OO (2010). Hypoglycemic and biochemical properties of *Cnestis ferruginea*. Afr. J. Tradit. Complement Altern. Med..

[37] Liu HY, Ivarsson E, Jonsson L, Holm L, Lundh T, Lindberg JE (2011). Growth performance, digestibility and gut development of broiler chickens on diets with inclusion of chicory (*Cichorium intybus*). Poult. Sci..

[38] Izadi H, Arshami J, Golian A, Raji MR (2013). Effects of chicory root powder on growth performance and histomorphometry of jejunum in broiler chicks. Vet. Res. Forum..

[39] Al-Jaff FK (2011). Effect of coriander seeds as diet ingredient on blood parameters of broiler chicks raised under high ambient temperature. Int. J. Poult. Sci..

[40] Brenes A, Roura E (2010). Essential oils in poultry nutrition: Main effects and modes of action. Anim. Feed. Sci. Technol..

[41] Przybilla P, Weiss J (1998). Herbal feed additives in pigs. DGS-Magazine.

[42] Rajeshwari U, Andallu B (2011). Medicinal benefits of coriander (*Coriandrum sativum* L). Spatula DD.

[43] Taraz Z, Shas SM, Samadi F, Ebrahimi P, Zerehdaran S (2015). Effect of chicory plant (*Cichorium intybus* L.) extract on performance and blood parameters in broilers exposed to heat stress with emphasis on antibacterial properties. Poult. Sci. J..

[44] Deans SG, Waterman PG, Hay RAM, Waterman PG (2011). Biological activity of volatile oils. Volatile Oil Crops, their Biology, Biochemistry and Production.

[45] Khubeiz MM, Abdelfettah M, Shirif AM (2020). Effect of coriander (*Coriandrum sativum* L.) seed powder as feed additives on performance and some blood parameters of broiler chickens. Open Vet. J..

[46] Rahimi S, Teymori Zadeh Z, Torshizi K, Omidbaigi R, Rokni H (2011). Effect of the three herbal extracts on growth performance, immune system, blood factors and intestinal selected bacterial population in broiler chickens. J. Agric. Sci. Tech..

[47] Abo Omar J, Hejazi A, Badran R (2016). Performance of broilers supplemented with natural herb extract. Open J. Anim. Sci..

[48] Panda K, Rama Rao SV, Raju LN, Shyam G, Sunder MV (2009). Effect of butyric acid on performance, gastrointestinal tract health and carcass characteristics in broiler chickens. Asian Aust. J. Anim. Sci..

[49] Yusrizal Y, Chen TC (2003). Effect of adding chicory fructans in feed on fecal and intestinal microflora and excreta volatile ammonia. Int. J. Poult. Sci..

[50] Somaieh N, Nobakht A, Safamehr A (2011). The effects of different levels of nettle *Urtica dioica* (Urticaceae) medicinal plant in starter and grower feeds on performance, carcass traits, blood biochemical and immunity parameters of broilers. Iran. J. Anim. Sci..

[51] Khubeiz MM, Shirif AM (2020). Effect of coriander (*Coriandrum sativum* L.) seed powder as feed additives on performance and some blood parameters of broiler chickens. Open Vet. J..

[52] Hosseinzadeh H, Qotbi A, Ahmad A, Seidavi A, Norris D, Brown D (2014). Effects of different levels of coriander (*Coriandrum sativum*) seed powder and extract on serum biochemical parameters, microbiota, and immunity in broiler chicks. Sci. World J..

[53] Cao FL, Zhang XH, Yu WW, Zhao LG, Wang T (2012). Effect of feeding fermented ginkgo biloba leaves on growth performance, meat quality, and lipid metabolism in broilers. Poult. Sci..

[54] Dhanapakiam P, Joseph JM, Ramaswany VK, Moorthi M, Kumar AS (2007). The cholesterol lowering property of coriander seeds (*Coriandrum sativum*) of action. J. Eviron. Biol..

[55] Aghazadeh A, Nabiyar E (2015). The effect of chicory root powder on growth performance and some blood parame-ters of broilers fed wheat-based diets. J. Appl. Anim. Res..

[56] Khoobani M (2020). Effects of dietary chicory (*Chicorium intybus* L.) and probiotic blend as natural feed additives on performance traits, blood biochemistry, and gut microbiota of broiler chickens. Antibiotics..

[57] Elson CE (1995). Suppression of mevalonate pathway activities by dietary isoprenoids: Protective roles in cancer and cardiovascular disease. J. Nutr..

[58] Kim M (2000). The water-soluble extract of chicory reduces cho lesterol uptake in gut-perfused rats. Nutr. Res..

[59] Bou R, Codony R, Tres A, Decker EA, Guardiola F (2009). Dietary strategies to improve nutritional value, oxidative stability, and sensory properties of poultry products. Crit. Rev. Food Sci. Nutr..

[60] Fattman CL, Chang LY, Termin TA, Petersen L, Enghild JJ (2003). Enhanced bleomycin-induced pulmonary damage in mice lacking extracellular superoxide dismutase. Free Radic. Biol. Med..

[61] Koracevic D, Koracevic G, Djordjevic V, Andrejevic S, Cosic V (2001). Method for the measurement of antioxidant activity in human fluids. J. Clin. Pathol..

[62] Ahmed SKH (2019). Egg yolk, fatty acids, blood parameters and some reproductive measurements of Japanese guial supplemented with chia seeds (*Salvia hispanice* L.). Int. J. Poult. Sci..

[63] Haq I, Ullah N, Bibi G, Kanwal S, Ahmad MS, Mirza B (2012). Antioxidant and cytotoxic activities and phytochemical analysis of *Euphorbia wallichii* root extract and its fractions. Iran J. Pharm. Res..

[64] Chang CC, Yang MH, Wen HM, Chern JC (2002). Estimation of total flavonoid content in propolis by two complementary colorimetric methods. J. Food Drug Anal..

[65] Brand-Williams W, Cuvelier ME, Berset C (1995). Use of free radical method to evaluate antioxidant activity. LWT-Food Sci. Technol..

[66] Benzie IF, Strain JJ (1996). The ferric reducing ability of plasma (FRAP) as a measure of “antioxidant power”: The FRAP assay. Anal. Biochem..

[67] Arnao MB, Cano A, Acosta M (2001). The hydrophilic and lipophilic contribution of total antioxidant activity. Food Chem..

[68] Chew YL, Goh JK, Lim YY (2009). Assessment of *in-vitro* antioxidant capacity and polyphenolic composition of selected medicinal herbs from Leguminosae family in Peninsular Malaysia. Food Chem..

[69] Liang Z, Cheng L, Zhong GY, Liu RH (2014). Antioxidant and antiproliferative activities of twenty-four *Vitis vinifera* grapes. PLoS ONE.

[70] Leggett GE, Westermann DT (1973). Determination of mineral elements in plant tissues using trichloroacetic acid extraction. J. Agric. Food Chem..

