# Author Correction: Analysis of the phytochemicals of *Coriandrum sativum* and *Cichorium intybus* aqueous extracts and their biological effects on broiler chickens

**DOI:** 10.1038/s41598-022-14645-5

**Published:** 2022-06-15

**Authors:** Hanaa S. S. Gazwi, Magda E. Mahmoud, Enas M. A. Toson

**Affiliations:** 1 Department of Agricultural Chemistry, Faculty of Agriculture, Minia University, El‑Minia, Egypt; 2 Department of Animal and Poultry Production, Faculty of Agriculture, Minia University, El‑Minia, Egypt

Correction to: *Scientific Reports* 10.1038/s41598-022-10329-2, published online 16 April 2022

In the original version of this Article Enas M. A. Toson was incorrectly affiliated with ‘Department of Agricultural Chemistry, Faculty of Agriculture, Minia University, El-Minia, Egypt’. The correct affiliation is listed below.

Department of Animal and Poultry Production, Faculty of Agriculture, Minia University, El-Minia, Egypt.

The original Article has been corrected.

